# Exploring the Capability of Cu-MoS_2_ Catalysts for Use in Electrocatalytic Overall Water Splitting

**DOI:** 10.3390/mi15070876

**Published:** 2024-07-03

**Authors:** Aviraj M. Teli, Rajneesh Kumar Mishra, Jae Cheol Shin, Wookhee Jeon

**Affiliations:** 1 Division of Electronics and Electrical Engineering, Dongguk University-Seoul, Seoul 04620, Republic of Korea; avteli.teli@gmail.com (A.M.T.); jcshin@dgu.ac.kr (J.C.S.); 2 Department of Physics, Yeungnam University, Gyeongsan 38541, Gyeongbuk, Republic of Korea; 3 Department of Semiconductor, Convergence Engineering, Sungkyunkwan University, Suwon 16419, Gyeonggi, Republic of Korea

**Keywords:** Cu-MoS_2_, HER, OER, overall water splitting, excellent stability

## Abstract

Herein, we prepare MoS_2_ and Cu-MoS_2_ catalysts using the solvothermal method, a widely accepted technique for electrocatalytic overall water-splitting applications. TEM and SEM images, standard tools in materials science, provide a clear view of the morphology of Cu-MoS_2_. HRTEM analysis, a high-resolution imaging technique, confirms the lattice spacing, lattice plane, and crystal structure of Cu-MoS_2_. HAADF and corresponding color mapping and advanced imaging techniques reveal the existence of the Cu-doping, Mo, and S elements in Cu-MoS_2_. Notably, Cu plays a crucial role in improving the hydrogen evolution reaction (HER) and oxygen evolution reaction (OER) of the Cu-MoS_2_ catalyst as compared with the MoS_2_ catalyst. In addition, the Cu-MoS_2_ catalyst demonstrates significantly lower overpotential (167.7 mV and 290 mV) and Tafel slopes (121.5 mV dec^−1^ and 101.5 mV dec^−1^), standing at −10 mA cm^−2^ and 10 mA cm^−2^ for HER and OER, respectively, compared to the MoS_2_ catalyst. Additionally, the Cu-MoS_2_ catalyst displays outstanding stability for 12 h at −10 mA cm^−2^ of HER and 12 h at 10 mA cm^−2^ of OER using chronopotentiaometry. Interestingly, the Cu-MoS_2_‖Cu-MoS_2_ cell displays a lower cell potential of 1.69 V compared with the MoS_2_‖MoS_2_ cell of 1.81 V during overall water splitting. Moreover, the Cu-MoS_2_‖Cu-MoS_2_ cell shows excellent stability when using chronopotentiaometry for 18 h at 10 mA cm^−2^.

## 1. Introduction

The rapidly growing urban landscape, defense industry, space divisions, and fossil fuel-based transportation sectors are causing global warming and disparities in the ecological system [1]. Therefore, the inescapable energy shortage and ecological concerns due to carbon emissions have unlocked an opportunity for widespread research on alternative energy sources [2,3]. Hydrogen, a widely distributed substance in the universe that is generated from various catalytic nanomaterials, is comprehensively accepted as a favorable energy source with which to substitute fossil fuels [4]. Furthermore, clean and green hydrogen can be achieved using the electrochemical splitting of water, without any further carbon secretion during the hydrogen and oxygen generation process [5]. Therefore, catalytic water splitting is considered an important and vital procedure for harvesting clean hydrogen energy from chemical energy [6], consisting of two half-cell reactions at the cathode (hydrogen evolution reaction, HER) and at the anode (oxygen evolution reaction, OER) [7]. Interestingly, OER provides electrons and protons for a reduction in half-reaction, creating major energy losses due to sluggish reaction kinetics [8]. Remarkably, the OER electrocatalysts with the greatest catalytic activities are precious metals and their oxides, namely Ru, RuO_2_, IrO_2_, and Ir [9]. However, the precious metal Pt is considered the standard electrocatalyst for HER, which is expensive and makes hydrogen generation overpriced [10]. Therefore, making progress in developing active, inexpensive, durable, and excellent conductive bifunctional catalysts for the HER and OER is one of the crucial tasks for several electrocatalytic water-splitting technologies. Curiously, numerous nanostructural materials have been studied to examine the capabilities of electrochemical water-splitting activities. 

Molybdenum disulfide (MoS_2_) is a prominent member of the transition metal dichalcogenide (TMD) family that has received noteworthy attention in recent years due to its intriguing physical, optoelectronic, mechanical, and magnetic features, and various potential applications [11]. As a nanolayered material, MoS_2_ displays an exclusive S-Mo-S sandwich structure, where molybdenum atoms are sandwiched between layers of sulfur atoms, exhibiting an n-type behavior [12,13]. This sandwich structure of the MoS_2_ divulges resilient edge active sites, which facilitate quick catalytic reaction kinetics, making it a more suitable candidate for electrocatalytic water-splitting technological developments [14]. MoS_2_ materials are multidimensional and outstanding and well recognized for their excellent optoelectronic, magnetic, mechanical, and ionic properties [15]. MoS_2_ is an extremely stable nanomaterial with weak van der Waals interactions between the layers and resilient in-plane covalent bonds that enable smooth mechanical exfoliation into thin sheets [16]. Interestingly, the exclusive features of MoS_2_, exhibited mainly when reduced to a few layers or a monolayer, facilitate its use in next-generation cutting-edge technologies. MoS_2_ exhibits distinct optical bandgap properties depending on its thickness. The electronic properties of the MoS_2_ are remarkable due to its switchable nature from an indirect bandgap (bulk form) to a direct bandgap (monolayer), which can be favorable for numerous applications. Moreover, MoS_2_ has an indirect bandgap of nearly 1.27 eV in the bulk form [17]. Besides, MoS_2_ displays a direct bandgap of about 1.90 eV in the few-layer or monolayer form [18]. The morphologies of MoS_2_ can differ significantly based on the synthesis techniques, which tune its properties and appropriateness in different applications. MoS_2_ can be synthesized in various forms, including nanotubes [19], nanosheets [20], nanoflowers [21], nanowires [14], and nanoribbons [22], each demonstrating unique edge structures, surface areas, and electrolysis water-splitting activities [23]. The morphologies of MoS_2_ play a decisive role in shaping its capabilities in various applications, such as the electrolysis of water [24], supercapacitors [25], field-effect transistors [26], batteries [27], perovskite light-emitting diode [28], and gas sensors [29]. Numerous production methods have been established to prepare MoS_2_ with precise morphologies and properties, which include hydrothermal qualities [30], solvothermal qualities [31], sol–gel applications [32], co-precipitation [33], pulse laser deposition [34], DC sputtering [35], and flame spray pyrolysis [36]. Interestingly, the above-discussed techniques offer distinctive benefits in terms of ease, scalability, and the capacity to modify the features of MoS_2_. Remarkably, chemical synthesis routes, such as hydrothermal and solvothermal approaches, have been extensively used for their capability to synthesize high-purity MoS_2_ with well-defined morphologies [37,38]. On the other hand, physical synthesis methods, such as RF sputtering and chemical vapor deposition, have been broadly utilized to prepare high-purity MoS_2_ thin films in vertically aligned morphologies with various applications [39,40]. Despite its prospective applications, MoS_2_ faces serious limitations, such as slow OER electrocatalytic reaction kinetics, high overpotential, and inadequate stability, which hamper its commercial applicability. Therefore, improving the catalytic performance of MoS_2_ is imperative for its practical application. Among the various doping materials investigated, copper (Cu) is a hopeful catalytic candidate for advancing the electrochemical activities of the MoS_2_. Therefore, the Cu-doped MoS_2_ (Cu-MoS_2_) boosts ionic interactions, magnifies the surface area, and enables electrical conduction, making it predominantly active in increasing electrocatalytic activities. Therefore, the doping of Cu into MoS_2_ can build chemically active defect states, which can increase overall conductivity, thereby regulating the overall water-splitting process. 

In this work, we investigate the electrocatalytic overall water splitting of the MoS_2_ and Cu-MoS_2_ catalysts. Fascinatingly, the MoS_2_ and Cu-MoS_2_ catalysts are prepared using a simple, scalable, and inexpensive solvothermal method. Attractively, Cu plays a dynamic role in ornamenting the electrocatalytic overall water-splitting properties of the Cu-MoS_2_ catalyst. Stimulatingly, the Cu-MoS_2_ catalyst shows a low overpotential and a low Tafel slope of HER and OER compared to the MoS_2_ catalyst. Fascinatingly, Cu-MoS_2_‖Cu-MoS_2_ cell shows low potential and excellent stability for 18 h at 10 mA cm^−2^. Moreover, the OER and HER mechanisms are also discussed to study the reaction process of hydrogen and oxygen generation.

## 2. Synthesis Methods

MoS_2_ and Cu-MoS_2_ were prepared using a straightforward one-step solvothermal process. Typically, 60 mL (1:1 ratio) of ethanol and DI water were mixed with 16 mg of C_2_H_5_NS and 4 mmol of Na_2_MoO_4_·2H_2_O, while being magnetically stirred to create a consistent solution. Furthermore, 2 mg of Cu was melted in 10 mL of DI water using a combination of magnetic stirring and sonication. Further, using magnetic stirring, 3 mL of the Cu precursor was gradually mixed dropwise into the 60 mL Mo and S precursor solution. The Mo and S precursor solution, mixed with Cu, was then shifted to an autoclave with a capacity of 100 milliliters. Additionally, a 2.2 cm × 3.0 cm portion of washed Ni-foam was put in the autoclave at 180 °C for 18 h to allow Cu-MoS_2_ to grow in situ on 3D Ni-foam. Finally, the Cu-MoS_2_ deposited on Ni-foam was washed with ethanol and DI water to remove the impurities and dehydrated at 95 °C under vacuum conditions for 15 h. Similarly, pure MoS_2_ was also synthesized using the above-discussed method with a Cu source.

The Cu-MoS_2_ morphology of the Ni-foam is meticulously scanned via a scanning electron microscope (SEM) obtained from S-4800 HITACHI, Ltd., Tokyo, Japan. Additionally, the structural, morphological, and elemental properties of the Cu-MoS_2_ are thoroughly studied via TEM, HRTEM, and HAADF with elemental mapping using the JEOL, JEM-2100F, JEOL Ltd., Tokyo, Japan. Moreover, the electrocatalytic activities of the MoS_2_ and Cu-MoS_2_ catalysts for electrocatalytic water splitting are rigorously tested using the electrochemical workstation VersaSTAT3 (Princeton Applied Research). The electrocatalytic performances of the MoS_2_ and Cu-MoS_2_ electrocatalysts are tested via a three-electrode arrangement in a 1.0 M KOH alkaline electrolyte. The MoS_2_ and Cu-MoS_2_ catalysts, Pt, and Ag/AgCl are used as working, counter, and reference electrodes, respectively. The overall water splitting is investigated in two-electrode arrangements in 1.0 M KOH, where MoS_2_ or Cu-MoS_2_ are used in both anode and cathode electrodes. The linear sweep voltammetry (LSV) of the MoS_2_ and Cu-MoS_2_ catalysts are studied at 5 mV s^−1^. Furthermore, the Tafel slopes of the MoS_2_ and Cu-MoS_2_ catalysts are accomplished from the LSV plot, using η=blog⁡j+a, where *b* is the Tafel slope, *j* is the current density, *a* is the transfer coefficient, and *η* is the overpotential. The recorded potential vs. Ag/AgCl is converted into the potential of the reversible hydrogen electrode (RHE) via the relation $E_{RHE}=E_{Ag/AgCl}^{o}+E_{Ag/AgCl}+0.059\times pH$.

## 3. Results and Discussions

**Figure 1:**
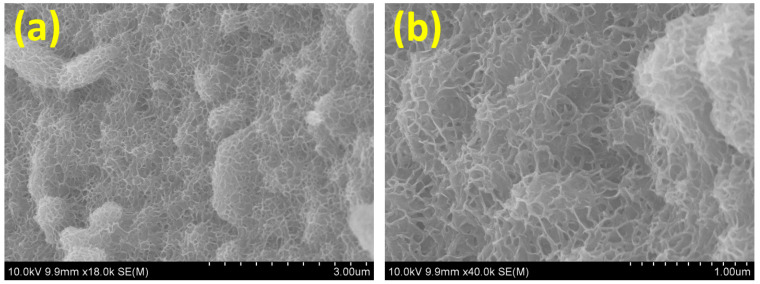
(**a**,**b**) The SEM pictures of the Cu-MoS_2_ on 3D Ni-foam at different magnifications.

Figure 1 elucidates the scanning electron microscopy (SEM) visuals of the Cu-MoS_2_. The shape of the Cu-MoS_2_ grown on the Ni-foam substrate is shown in Figure 1a,b at different magnifications, exploring the different orientations of the nanolayers. Further, the nanolayered morphology of Cu-MoS_2_ is described in Figure 1a,b, where the outermost portions of the nanolayers can be seen in various directions, enabling a greater chance for engagement with the alkaline electrolyte, which can be beneficial for improving the electrocatalytic water-splitting activities. Also, the surface area of Cu-MoS_2_ can be significantly expanded by these multilayered architectures, and thus, these unique nanolayer structures create ideal sites of interaction on the Cu-MoS_2_ surface for hydrogen and oxygen evolution reaction procedures. Moreover, Cu-doping in Cu-MoS_2_ boosts the number of engaged reaction sites and their area of interface with the KOH electrolyte, which can enhance the catalytic capabilities of the hydrogen and oxygen evolution reaction. In addition, this unique Cu-MoS_2_ morphology can also promote the transfer of electrons due to the highly conductive nature of Cu in the Cu-MoS_2_. However, the surface structure of the Cu-MoS_2_ also indicates that it is able to transfer hydrogen and oxygen molecules and improve adsorption and desorption to accelerate kinetics for the development of hydrogen and oxygen in overall water splitting. 

**Figure 2:**
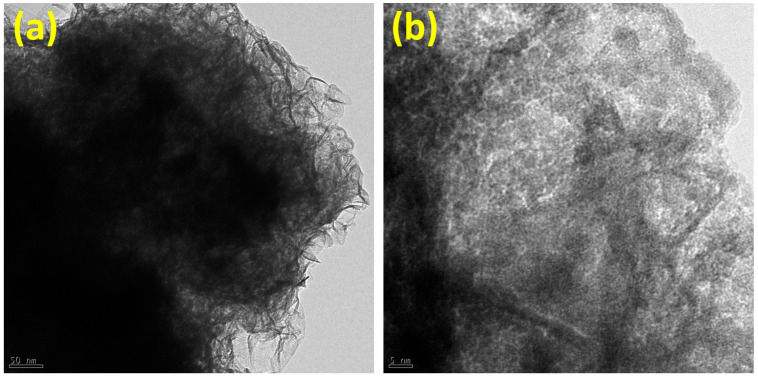
(**a**,**b**) TEM images of the Cu-MoS_2_ at different magnifications.

**Figure 3:**
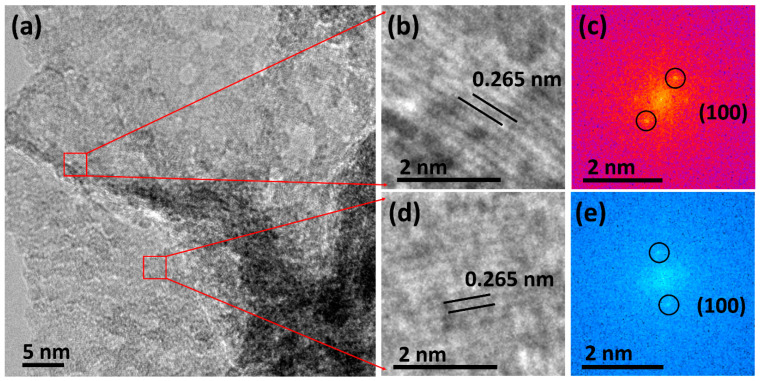
(**a**) HRTEM image, (**b**,**d**) HRTEM images from the enlarged area, and corresponding (**c**,**e**) FFT patterns of the Cu-MoS_2_.

**Figure 4:**
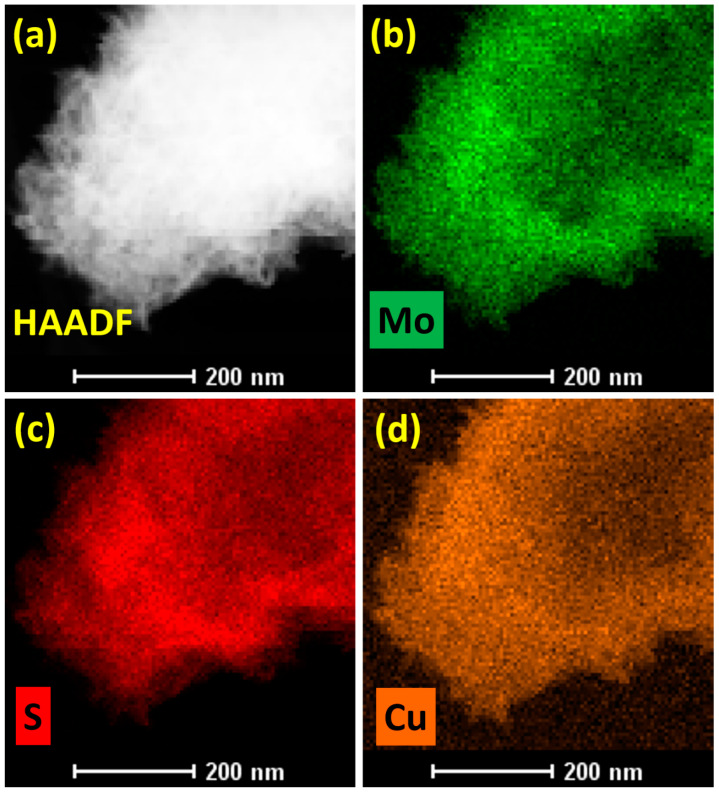
(**a**) HAADF image and elemental mapping of (**b**) Mo, (**c**) S, and (**d**) Cu elements of the Cu-MoS_2_.

Additionally, Figure 2a,b depict the transmission electron microscopy (TEM) images of the Cu-MoS_2_ to further study morphology on the nanoscale at different magnifications. It is observed that the synthesized Cu-MoS_2_ illustrates the agglomerated nanolayers. It is also important that the nanoscale shape of the Cu-MoS_2_ plays a significant role in the creation of the catalytically active sites on the catalyst surface, which can be beneficial for overall water-splitting applications. Interestingly, crumbled nanolayers of Cu-MoS_2_ also offer more exposure area for the KOH electrolyte during oxygen, hydrogen, and overall water-splitting tests. Consequently, it is expected that the TEM images will support the SEM results, as depicted in Figure 1. Furthermore, the structural examination of the Cu-MoS_2_ is studied using high-resolution TEM (HRTEM) and FFT, as portrayed in Figure 3a–e. Figure 3a shows the HRTEM image of the Cu-MoS_2_, illustrating several small crystallites, which show the lattice fringes of the Cu-MoS_2_. Interestingly, these small crystallites merge together to form the nanolayers of Cu-MoS_2_, which have a significant impact on the creation of various lattice strains in the Cu-MoS_2_ nanolayers. Figure 3b,d display the zoomed portion of the HRTEM image of Cu-MoS_2_ to reveal 0.265 nm lattice spacing. Figure 3c,e present the FFT images of the Cu-MoS_2_ from the HRTEM area, as shown in Figure 3b,d. The FFT images of the Cu-MoS_2_ show the lattice plane (100) corresponding to 0.265 nm lattice spacing. Moreover, the lattice spacing (0.265 nm) and lattice plane (100) of the synthesized Cu-MoS_2_ are consistent with those of the JCPDS card no. 37-1492, which confirms the hexagonal crystal structure [41]. In addition, to verify the Cu-doping, color mapping of the Cu-MoS_2_ is performed and the results are shown in Figure 4a–d. Figure 4a reveals the high-angle annular dark field-scanning (HAADF) image of the Cu-MoS_2_, which is used for the color mapping of Mo, S, and Cu elements. Figure 4b–d exhibit the color mapping of Cu, Mo, and S elements, which confirms the doping of Cu atoms in the Cu-MoS_2_.

**Figure 5:**
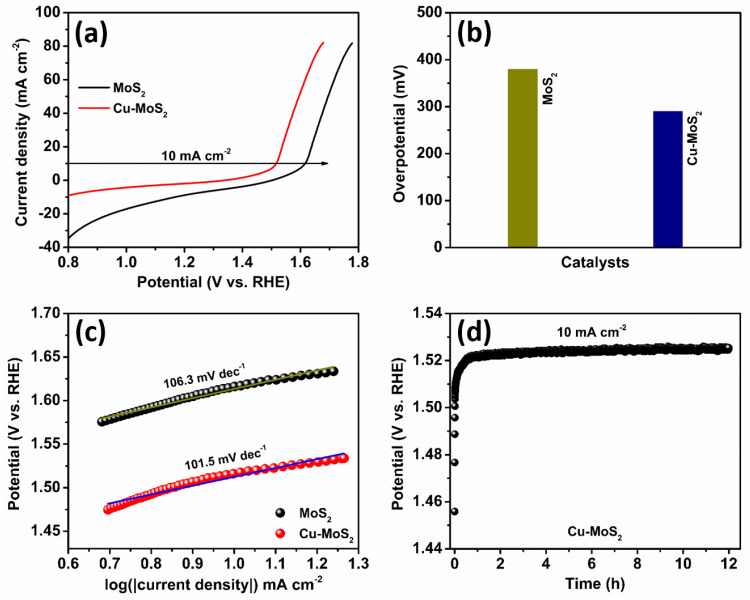
Electrocatalytic oxygen evolution reaction (OER). (**a**) LSV plots, (**b**) overpotential, and (**c**) Tafel plots of the MoS_2_ and Cu-MoS_2_ catalysts. (**d**) Stability test of the Cu-MoS_2_ catalyst at 10 mA cm^−2^ for 12 h.

The oxygen evolution reaction (OER) has a high energy barrier for its four-electron transfer mechanism, hindering the rate of electrocatalytic reaction kinetics. The electrochemical performance of the MoS_2_ and Cu-MoS_2_ electrocatalysts for OER is studied by a three-electrode arrangement in a 1.0 M KOH alkaline medium. Figure 5a depicts the linear sweep voltammetry (LSV) plots, comparing the characteristics of the MoS_2_ and Cu-MoS_2_ catalysts at 5 mV s^−1^. The LSV plots noticeably elucidate that the Cu-MoS_2_ catalyst reveals remarkably higher OER performances than the MoS_2_ catalyst. Moreover, Figure 5b divulges the overpotentials of the Cu-MoS_2_ and MoS_2_ electrocatalysts. Stimulatingly, the Cu-MoS_2_ catalyst specifies a smaller overpotential of 290 mV than the MoS_2_ catalyst of 380 mV. This reduction in the overpotential of the Cu-MoS_2_ electrocatalyst is due to the doping of Cu, which improves electron transport and favorable adsorption energies and reduces the energy barrier during the OER process [42,43]. Figure 5c elucidates the Tafel plots of the Cu-MoS_2_ and MoS_2_ electrocatalysts to examine the reaction rate kinetics. It is observed that the Tafel slope of the Cu-MoS_2_ electrocatalyst is 101.5 mV dec^−1^, which is smaller than that of the MoS_2_ electrocatalyst at 106.3 mV dec^−1^. In addition, Figure 5d discloses results of a stability test performed to estimate the robustness of the Cu-MoS_2_ catalyst. It is observed that the initial potential of stability of the Cu-MoS_2_ catalyst is 1.52 V, which upsurges to 1.54 V after 12 h of the chronopotentiometry test at a current density of 10 mA cm^−2^. The outstanding stability of the Cu-MoS_2_ electrocatalyst is due to its various features, such as the strong interaction between the Cu-doping atom and host MoS_2_, the high surface area, its low susceptibility to structural breakdown, and the decreased deactivation of the active sites on the Cu-MoS_2_ electrocatalyst surface [44,45]. The structural stability, such as mechanical stress; well-created active sites; lack of significant degradation during the long-term stability test for 12 h; and excellent thermal stability of the nanolayered Cu-MoS_2_ catalyst were significant [46,47]. 

**Figure 6:**
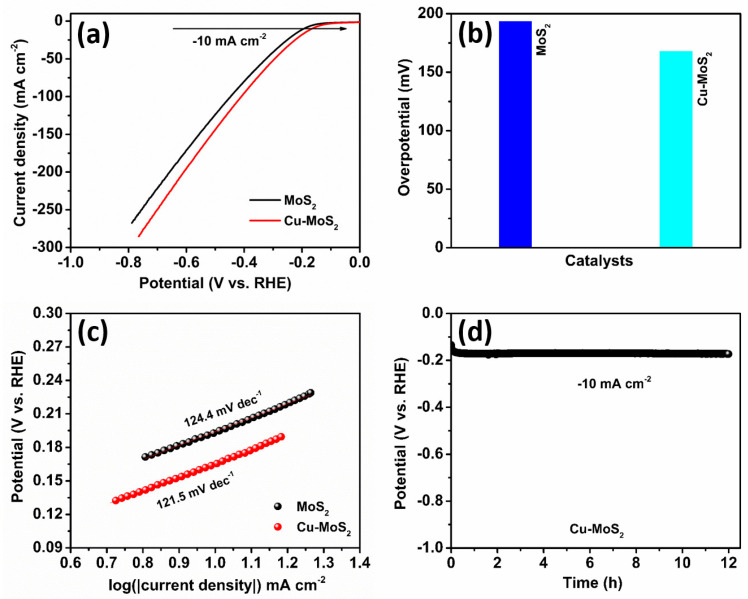
Electrocatalytic hydrogen evolution reaction (HER). (**a**) LSV plots, (**b**) overpotential, and (**c**) Tafel plots of the MoS_2_ and Cu-MoS_2_ catalysts. (**d**) Stability test of the Cu-MoS_2_ catalyst at −10 mA cm^−2^ for 12 h.

The HER catalytic activities of the Cu-MoS_2_ and MoS_2_ electrocatalysts are investigated using the simple three-electrode process in an alkaline 1.0 M KOH. Figure 6a displays the linear sweep voltammetry (LSV) plots of the MoS_2_ and Cu-MoS_2_ catalysts at a scan rate of 5 mV s^−1^. As depicted in the LSV plots in Figure 6a, the Cu-MoS_2_ catalyst unveils superior hydrogen evolution activities to the MoS_2_ catalyst. Interestingly, the onset potential of the Cu-MoS_2_ catalyst is lower than that of the MoS_2_ catalyst, which necessitates low energy use to initiate the electrocatalytic hydrogen evolution reaction [48,49]. Therefore, it is expected that the Cu-MoS_2_ catalyst can offer a greater rate of hydrogen generation than the MoS_2_ catalyst at the same applied potential. Further, Figure 6b presents overpotential plots of the MoS_2_ and Cu-MoS_2_ catalysts, providing vital insight into their electrocatalytic efficiency. The Cu-MoS_2_ catalyst illustrates a lower overpotential of 167.7 mV than the MoS_2_ catalyst, which has one of 193.4 mV. It is supposed that the Cu-doping might be responsible for the increased presence of active sites on the Cu-MoS_2_ electrocatalyst surface [50]. Also, Cu-doping in MoS_2_ can create structural changes in the electronic properties of the Cu-MoS_2_ catalyst, leading to reduced overpotential [42]. Fascinatingly, Tafel slopes are vital factors in electrocatalytic hydrogen evolution reactions, offering insightful indications regarding the reaction kinetics and mechanism [51]. Figure 6c elucidates the Tafel plots of the Cu-MoS_2_ and MoS_2_ electrocatalysts to examine their reaction rate kinetics and mechanisms. It is observed that the Tafel slope value of the Cu-MoS_2_ electrocatalyst is lower, at 121.5 mV dec^−1^, than that of the MoS_2_ catalyst at 124.4 mV dec^−1^. This reduces the Tafel slope value of the Cu-MoS_2_ catalyst, suggesting that low overpotential is necessary to reach −10 mA cm^−2^. Interestingly, the doping of the Cu atom can increase the electron transfer capability of the Cu-MoS_2_ electrocatalyst and further lower the energy barrier during the HER process. Moreover, Figure 6d reveals the results of a stability test of the Cu-MoS_2_ catalyst, performed to inspect its durability at −10 mA cm^−2^. Using the chronopotentiometry test, it is observed that the initial potential of the Cu-MoS_2_ electrocatalyst is 166.8 mV, and it reaches up to 173.2 mV after 12 h of stability. Excitingly, it can be the intrinsic properties of the 2D-layered Cu-MoS_2_ catalyst, such as outstanding chemical stability, surface area, and resistance to corrosion, which influence the adsorption and desorption process during the 12 h stability test [52,53,54].

**Figure 7:**
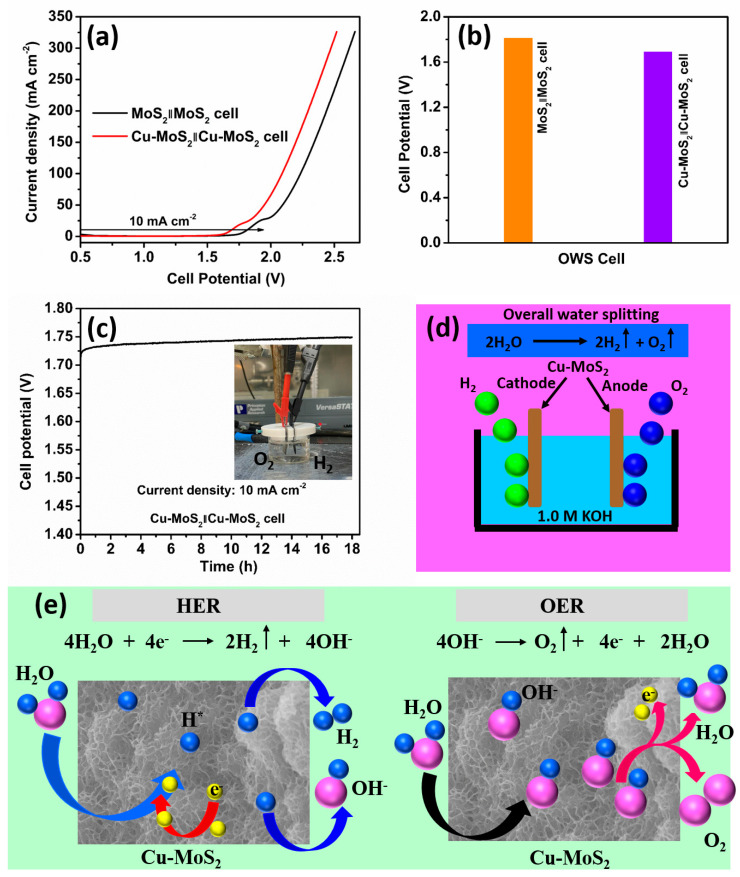
(**a**) LSV plots. (**b**) Cell potentials of the MoS_2_‖MoS_2_ cell and Cu-MoS_2_‖Cu-MoS_2_ cell. (**c**) Stability of the Cu-MoS_2_‖Cu-MoS_2_ cell at 10 mA cm^−2^ (inset—optical photograph) and (**d**) schematic presentation of the overall water-splitting mechanism of the Cu-MoS_2_‖Cu-MoS_2_ cell. (**e**) Schematic depiction of the impact of the HER and OER mechanisms on the Cu-MoS_2_ catalyst surface.

Further, we investigated the electrocatalytic activities of the MoS_2_‖MoS_2_ cell and Cu-MoS_2_‖Cu-MoS_2_ cell in a two-electrode arrangement (overall water splitting) in 1.0 M KOH alkaline. Figure 7a displays the linear sweep voltammetry (LSV) plots of the MoS_2_‖MoS_2_ cell and Cu-MoS_2_‖Cu-MoS_2_ cell at 5 mV s^−1^ in overall water splitting. It is observed that Cu-doping in the MoS_2_ plays a significant role in the shift of the LSV curve toward the low-potential side. Figure 7b displays the cell potential in the overall water splitting of the MoS_2_‖MoS_2_ cell and Cu-MoS_2_‖Cu-MoS_2_ at 10 mA cm^−2^. Fascinatingly, the Cu-MoS_2_‖Cu-MoS_2_ cell shows a small cell potential of 1.69 V compared to the MoS_2_‖MoS_2_ cell’s potential of 1.81 V in overall water splitting. The low cell potential of the Cu-MoS_2_‖Cu-MoS_2_ cell compared with MoS_2_‖MoS_2_ cell is ascribed to the synergistic influence of Cu-doping in the host MoS_2_. Curiously, the stability of the MoS_2_‖MoS_2_ cell is vital for everyday applications in overall water-splitting applications, where enduring practical productivity is essential. Figure 7c parades the stability results after 18 h of processing the Cu-MoS_2_‖Cu-MoS_2_ cell at a 10 mA cm^−2^ current density in an overall water-splitting process. Using the chronopotentiometry test, it is observed that the cell potential upsurges from 1.726 V to 1.75 V during 18 h of testing at 10 mA cm^−2^. The inset in Figure 7c shows an optical photograph of the overall water splitting. Remarkably, various factors, including electrolyte composition, conductivity, ion exchange, corrosiveness, impurity atoms, and pH, affect the overall water-splitting durability of the Cu-MoS_2_‖Cu-MoS_2_ cell during the 18 h chronopotentiometry test. Furthermore, the doping of the Cu atom in the Cu-MoS_2_ catalyst its reduces structural degradation over 18 h of stability and enhances its surface chemistry, which can decrease the corrosion of the catalyst, creating excellent synergy between Cu-atom and MoS_2_ in the Cu-MoS_2_ catalyst, improving charge-transfer, and reducing the energy required to complete the overall water-splitting reaction [55,56]. 

Further, in the Cu-MoS_2_‖Cu-MoS_2_ cell, the water-splitting mechanism implicates both the hydrogen evolution reaction (HER) and oxygen evolution reaction (OER). The HER mechanism is elaborated in the following Equations (1)–(3) [41,57] and illustrated in Figure 7e.

Volmer process:

(1) $$H_{2}O+e^{-}+*\;\to H^{*}+OH^{-}$$

Heyrovsky process:

(2) $$H_{2}O+e^{-}+H^{*}\to H_{2}+OH^{-}+*$$

Tafel process:

(3) $$H^{*}+H^{*}\to H_{2}$$

Figure 7e shows the HER mechanism, which follows the Equations (1) and (2) because the Tafel slope value lies in the range of the Heyrovsky process.

Fascinatingly, the oxygen evolution reaction (OER) is sluggish, and it needs to be improved using different variations in the electrocatalysts. Generally, the OER is the adsorption four-electron transfer process. The OER takes place in various steps in alkaline electrolytes, as shown in the following, Equations (4)–(7) [58,59].

(4) $$M+OH^{-}\to M-OH+e^{-}$$

(5) $$M+OH^{-}\to M-OH+e^{-}$$

(6) $$MO+OH^{-}\to MOOH+e^{-}$$

(7) $$MOOH+OH^{-}\to M+O_{2}+H_{2}O+e^{-}$$

In Equation (4), the adsorption of *OH^–^* with the active site *M* onto the Cu-MoS_2_ electrocatalyst’s surface generates *OH* and an electron. In Equation (5), the interaction between *OH* and *OH^–^* of the Cu-MoS_2_ electrocatalyst surface and then the bond-breaking process produce water molecules, oxygen, and an electron. Moreover, the second adsorption process is discussed in Equation (6), which illustrates the intermediate state *OOH*, making bonds with active site *M* on the Cu-MoS_2_ electrocatalyst’s surface. Furthermore, the intermediate state *OOH*, adsorbed onto the active site *M* on the Cu-MoS_2_ surface, further interacts with *OH*, which produces water molecules, creates oxygen, and releases an electron as discussed in Equation (7). Figure 7d,e exposes the schematic illustration of the overall water splitting of the Cu-MoS_2_‖Cu-MoS_2_ cell. Interestingly, the graphic depiction in Figure 7d,e explores the understanding of the electrocatalytic developments occurring at both the anode (Cu-MoS_2_) and cathode (Cu-MoS_2_) electrodes in 1.0 M KOH. Unusually, protons from electrolytes are reduced at the cathode (Cu-MoS_2_) electrode to produce hydrogen energy. However, water molecules are oxidized at the anode (Cu-MoS_2_) electrode to produce oxygen, protons, and electrons. Moreover, Table 1 lists the electrocatalytic results reported works in the literature for comparison with the present study. The Cu-MoS_2_ catalyst is observed to be better than or comparable to the reported results. 

## 4. Conclusions

In conclusion, the Cu-MoS_2_ catalyst, which is synthesized by the simple and scalable solvothermal method, shows remarkable electrocatalytic activities in the overall water-splitting application. The Cu-MoS_2_ catalyst depicts low overpotential and small Tafel slopes during HER and OER. We also investigate whether the Cu-MoS_2_ catalyst elucidates the outstanding stability of OER (at 10 mA cm^−2^) and HER (at −10 mA cm^−2^) in a three-electrode setup. The Cu-MoS_2_‖Cu-MoS_2_ cell illustrates excellent stability for 18 h and a small cell potential of 1.69 V at 10 mA cm^−2^. Therefore, it is concluded that the Cu-MoS_2_‖Cu-MoS_2_ cell shows enhanced electrocatalytic activities due to Cu doping, resulting in a lower cell potential and outstanding stability in overall water splitting, which is crucial for practical applications in renewable energy technologies. Furthermore, variations in the Cu-doping concentration, the Mo and S precursor concentrations, and the solvothermal reaction temperature and time need to be further optimized to tailor the morphology and properties of the Cu-doped MoS_2_ for various applications, such as in sensors, memory devices, supercapacitors, batteries, and photocatalysis.

## Figures and Tables

**Table 1:** Comparative study of the electrocatalytic activities of the Cu-MoS_2_ catalyst with other results reported from the literature.

S. No.	Catalysts	Electrolyte	Overpotential	Stability	Ref.
Oxygen Evolution Reaction					
1	MoS_2_ quantum dots	1.0 M KOH	370 mV (10 mA cm^−2^)	2 h (10 mA cm^−2^)	[60]
2	MoS_2_ nanosheets wrapped MOF-based Co_3_O_4_	1.0 M KOH	230 mV (10 mA cm^−2^)	13 h (10 mA cm^−2^)	[61]
3	Metal–organic-framework-derived Co_9_S_8_-MoS_2_	1.0 M KOH	270 mV (10 mA cm^−2^)	24 h (10 mA cm^−2^)	[44]
4	MoS_2_-based hybrid with N-doped carbon-wrapped CoFe alloy	1.0 M KOH	337 mV (10 mA cm^−2^)	24 h (10 mA cm^−2^)	[62]
5	Cu-MoS_2_	1.0 M KOH	290 mV (10 mA cm^−2^)	12 h (10 mA cm^−2^)	This work
Hydrogen Evolution Reaction					
6	Cu-MoS_2_/NiS_2_	1.0 M KOH	105 mV (−10 mA cm^−2^)	--	[42]
7	W-1T MoS_2_-15	0.5 M H_2_SO_4_	292 mV (−10 mA cm^−2^)	14 h (−10 mA cm^−2^)	[63]
8	Mix-phased 1 T/2 H MoS_2_	1.0 M KOH	145 mV (−10 mA cm^−2^)	24 h (−10 mA cm^−2^)	[64]
9	AC/MoS_2_–F	0.5 M H_2_SO_4_	136 mV (−10 mA cm^−2^)	24 h (−10 mA cm^−2^)	[65]
10	Cu-MoS_2_	1.0 M KOH	167.7 mV (−10 mA cm^−2^)	12 h (−10 mA cm^−2^)	This work
Overall water splitting					
11	CoS/MoS_2_||CoS/MoS_2_	1.0 M KOH	1.61 V (cell potential) (10 mA cm^−2^)	12 h (10 mA cm^−2^)	[66]
12	MoS_2_-CoFeLDH/NF|| MoS_2_-CoFeLDH/NF	1.0 M KOH	1.55 V (cell potential) (10 mA cm^−2^)	48 h (10 mA cm^−2^)	[23]
13	MoS_2_/NiFe_2_O_4_|| MoS_2_/NiFe_2_O_4_	1.0 M KOH	1.69 V (cell potential) (10 mA cm^−2^)	--	[67]
14	1T-MoS_2_/Ni_3_S_2_/LDH|| 1T-MoS_2_/Ni_3_S_2_/LDH	1.0 M KOH	1.55 V (cell potential) (10 mA cm^−2^)	20 h (10 mA cm^−2^)	[68]
15	Cu-MoS_2_||Cu-MoS_2_	1.0 M KOH	1.69 V (cell potential) (10 mA cm^−2^)	18 h (10 mA cm^−2^)	This work

## Data Availability

The original contributions presented in the study are included in the article, further inquiries can be directed to the corresponding authors.
